# Toward stressor-free stress estimation: The integrated information theory explains the information dynamics of stress

**DOI:** 10.1016/j.isci.2024.110583

**Published:** 2024-07-26

**Authors:** Takayuki Niizato, Yuta Nishiyama, Yuta Oka, Poe Thinzar Aung, Shusaku Nomura

**Affiliations:** 1 Department of Intelligent Interaction Technologies, Institute of Systems and Information Engineering, University of Tsukuba, Tsukuba, Ibaraki, Japan; 2 Department of Information and Management Systems Engineering, Nagaoka University of Technology, Nagaoka, Niigata, Japan

**Keywords:** Neuroscience, Psychology, computational neuroscience

## Abstract

Recent neurological studies have revealed several detailed stress mechanisms. However, the latent variables behind stress study still interpret stress responses as difficult. Therefore, we propose a stressor-free method of stress evaluation using Integrated Information Theory (IIT) to address these issues. We conducted experiments inducing acute stress responses against tasks with three levels of difficulty, easy, moderate, and difficult, to verify the IIT in stress study. The moderate condition was related to active coping with stress. By contrast, the easy and difficult conditions are related to passive stress coping. Especially, the easy condition seemed to cause boredom. Our results revealed that the degree of entangled system fluctuation was associated with the subjective ratings for the tasks. Interestingly, our method could also evaluate stress as a state of boredom that had not received much research attention. Our method can be an alternative stress estimation method to overcome the latent variable problem.

## Highlights

• IIT quantifies entangled information among bio-signals relevant to stress responses
• IIT index distinguishes responses to tasks with different levels of difficulty
• IIT index is associated with subjective ratings reflecting psychological valence
• IIT index identifies responses derived from the state of boredom

## Graphical abstract

**Figure:**
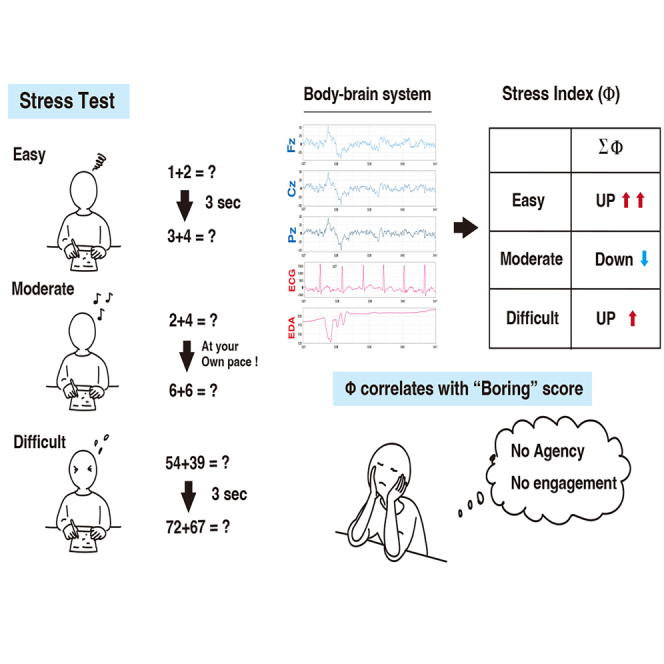

## Introduction

The phenomenon of stress seems to be subjective (e.g., feeling) and also objective (e.g., neuroscience, autonomic system, and immune system).^1^ It is a complex concept derived from a mind-body problem and has been studied in an interdisciplinary field ranging from physiology^2^ to sociology.^3^^,^^4^^,^^5^^,^^6^ Classically, the stress response was explained as a tuning of the autonomic nervous system.^7^ From this perspective, the stress response results from adaptively coping with various stimuli that disrupt the body’s balance.^2^^,^^8^ A trigger that induces the stress response is called a stressor (it can be an external/internal event in the system).^9^ The physiological system responds to stress via the hypothalamic-pituitary-adrenal (HPA) axis and sympathetic-adrenal-medullary (SAM) axis. The SAM axis relates to acute stress responses throughout the autonomic nervous system (e.g., freeze and fight-fright) responses).^1^^,^^8^^,^^10^ The HPA axis is associated with a relatively long-term hormone-induced stress response. Many inhibition and excitation processes occur around the paraventricular hypothalamic nucleus.^11^ These brain networks result in adaptive/maladaptive behaviors.

The main problem in stress research is an inconsistency between physiological responses and psychological assessments due to latent variables. Campbell has pointed out that the correlation between cortisol (i.e., a representative measure of the HPA axis^8^^,^^12^) and psychological stress is only about 25%.^13^ Similarly, measures based on the SAM axis (e.g., heart rate^14^ and skin conductance^15^) often do not match.^16^^,^^17^^,^^18^ Moreover, activities on the SAM and HPA axes can vary with age,^19^^,^^20^ gender^20^, and personality.^18^^,^^21^

Uncontrollable external variables, such as task engagement and individual predispositions, can further complicate the results of measurements.^13^^,^^22^ For example, flow experience and boredom as task engagement forms show both a stress response and a non-stress response. Boredom may not alter cortisol levels but can increase heart rate and skin conductance^23^ (but some researchers provide opposite views^24^^,^^25^). Flow experience shows mildly increasing cortisol and heart rate variability^26^ (but decreasing HRV has also been reported^27^), but sometimes it is hard to discriminate between overload and overload.^28^ Therefore, relying only on the HPA measure or the SAM measure is insufficient to evaluate the stress state.

Furthermore, pathological conditions (e.g., ADHD or depression) also show different stress responses compared with healthy participants. In the case of ADHD, for instance, Lackschewitz et al. have suggested that the correlation between cortisol and heart rate is the inverse of that in healthy individuals.^29^ The SAM-axis of depressive individuals can show different responses due to their prolonged stress state.^21^^,^^30^ In addition, recent research has also illuminated the different stress responses within virtual reality environments.^31^^,^^32^ Therefore, even when faced with the same stimulus, the stress responses of people with pathological conditions are likely to be based on unusual stress coping strategies.^22^

In this study, we propose that the inconsistent stress responses estimated by multiple physiological data due to the latent variables should not be viewed as flaws in stress research but as essential aspects of the stress phenomenon. Physiological datasets obtained from different methods of measurement should be regarded as inseparable fluctuations of a single integrated system. Thus, we introduce Integrated Information Theory (IIT) to the stress study. As mentioned later in discussion, our method to evaluate stress responses would offer a comprehensive understanding of stress without assuming the latent variables.

Firstly, the IIT would be able to quantify the stress response as a single system and estimate the subjective feelings accompanying the response, contributing to stress research. Although IIT primarily quantifies the degree of consciousness,^33^^,^^34^^,^^35^^,^^36^ the basic concept provides an information-theoretic measure for any system integration.^37^^,^^38^^,^^39^ The time series of datasets in IIT are not treated independently but are considered entangled fluctuations of a whole system.^34^ The central measure of IIT is known as Φ. This value represents the system’s irreducibility to the parts.^33^^,^^34^^,^^35^ According to Tononi et al., Φ measures the minimum amount of information lost when the system is divided into two parts.^33^ Suppose the loss of information is zero (Φ=0) when a system is divided into two. In that case, this value implies that the system consists of two independent systems unaffected by the division, which means the system is reducible to its components (i.e., two independent subgroups). Consequently, Φ indicates the degree of integrity, which cannot be reduced to the parts. In this study, our dataset comprises an electrocardiogram (ECG) and electrodermal activity (EDA) in addition to an electroencephalogram (EEG). Therefore, Φ does not simply quantify the amount of consciousness suggested by previous applications but rather represents the entangled fluctuation of a system against a specific stimulus. Particularly in a stressful situation, Φ can be understood as a measure of entangled interactions among system components.^34^^,^^36^ The entire system responds to the stimulus, regardless of its type. The responses of individual datasets take on meaning in the whole context. However, the entangled information process does not directly represent but is relevant to subjective feelings through reflection. Indeed, previous studies have shown that the subjective strength of perceptual illusion, the so-called rubber hand illusion,^40^ is associated with Φ calculated from several physiological datasets, suggesting that the value of Φ captures the subjective feeling of “ownership.”^41^

Secondly, by applying IIT to stress research, we would be able to leverage existing research findings effectively and thereby provide a more comprehensive understanding of stress and its mechanisms. IIT offers a significant shift in the traditional stimulus-response paradigm, presenting a paradigm to ask, “What system is?”^37^ This theoretical shift, unique to IIT, focuses solely on entangled system fluctuations, thereby eliminating the need to consider the types of stressors or the presence of latent variables. In this context, our measurements facilitate the introduction of a stressor-free methodology in stress response (note that “stressor-free” refers to the ability to measure stress without the need to consider the types of stressors).

**Figure 1:**
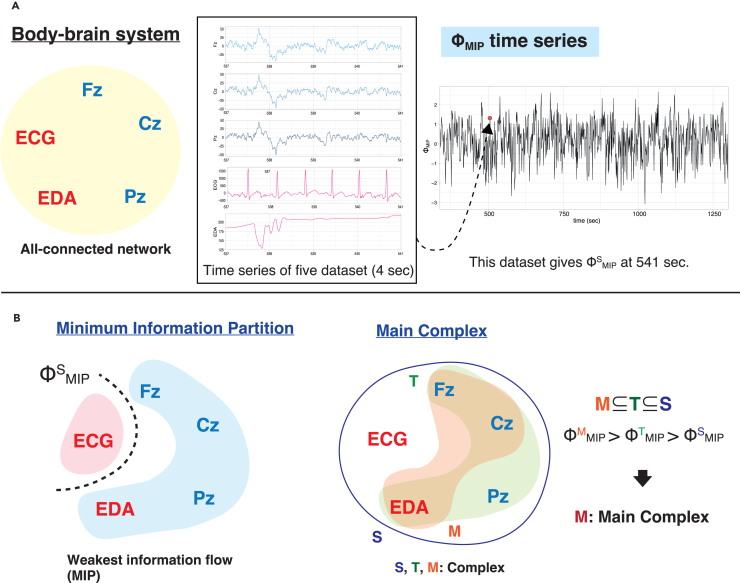
Schematic diagram of IIT application and IIT concepts (A) Schematic diagram of IIT application. We set the whole system S = {Fz, Cz, Pz, ECG, EDA}. $\Phi_{\text{MIP}}^{S}$ at time t = 541 (s) is computed from five types of time series. (B) Concepts of IIT. LEFT: Minimum information partition (MIP) is located between {ECG} and {Fz, Cz, Pz, EDA}. $\Phi_{\text{MIP}}^{S}$ is the loss of information led by MIP. RIGHT: Let S,T,M be complex. If M⊆T⊆S and $\Phi_{\text{MIP}}^{M}>\Phi_{\text{MIP}}^{T}>\Phi_{\text{MIP}}^{S}$, then M is a main complex.

To verify the applicability of the IIT to the stress study, we conducted experiments on the acute stress response induced by the calculation tasks.^42^^,^^43^ Experimental conditions were set up at three levels of difficulty: easy, moderate, and difficult (see the section experimental conditions for details). In addition, we assumed that participants would have different stress-coping strategies according to whether the task progress was either controllable (i.e., active coping) or uncontrollable (i.e., passive coping).^44^ In the moderate condition, the task was a medium stressor, and participants actively coped with it in their own time. In the difficult condition, the task was a high stressor, and participants passively coped because it took too short time to complete it. In the easy condition, the task could be less stressful, but participants would still passively cope with the task by having nothing to do. During the task, we recorded both brain activity (EEG) and internal bodily states (ECG and EDA), as well as the previous study.^41^ In combination with IIT analysis of those physiological data and psychological measurements (Figure 1), we show that the sum of the $\Phi_{\text{MIP}}$ (the degree of the entangled system fluctuation) correlates with the subjective ratings, such as excitement and boring. Our results suggest that IIT can estimate subjective feelings from the objective stress dataset even without the explicit stressor, as introduced in easy task.

## Results

### Minimum information partition for the stress response

First, we investigated the minimum information partition (MIP) of the entire system (i.e., *S* = {Fz, Cz, Pz, ECG, EDA}). The MIP is a system partition, making the system’s integrity minimal. Two measures exist: the MIP cut location and $\Phi_{\text{MIP}}^{S}$, where S represents a set consisting of all nodes of a system. As the MIP cut indicates the weakest link to the remaining elements, the cut location suggests the disconnected segments of the system driving the stress response. $\Phi_{\text{MIP}}^{S}$ denotes the degree of entangled information in a given network. We applied the *Z* score of $\log\;\Phi_{\text{MIP}}^{S}$ to reduce data variance. We defined the pre-task condition as the baseline. Therefore, the mean and variance of the $z{\left(\log\;\Phi_{\text{MIP}}^{S}\right)}_{t}$ of the pre-task region (t∈[1,537]) were set to 0 and 1, respectively.

**Figure 2:**
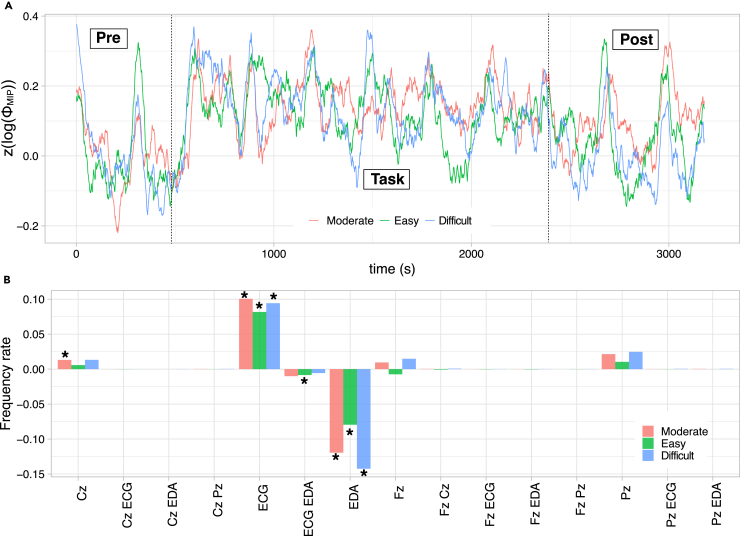
$z\left(\log\;\Phi_{\text{MIP}}^{S}\right)$ and its MIP cut locations (A) The time series of $z\left(\log\;\Phi_{\text{MIP}}^{S}\right)$ for the three conditions. After the pre-task state (around 580 s), $z\left(\log\;\Phi_{\text{MIP}}^{S}\right)$ rises for all conditions. The disturbances (i.e., quasi-peaks) were observed every 500 s, resulting from the experimental protocol of saliva collection (note that hormonal data was not the target of this study). (B) The pre-task frequency was subtracted from the mean MIP location frequency (18 participants) during the task. The value is expected to be 0 when the frequency does not change from the pre-task to the task. The asterisk represents the statistically significant frequency compared with 0 (results from *t*-test if normative assumption is satisfied, otherwise, Mann–Whitney U test: p<0.05).

Figure 2A shows the time series for each condition. The $z\left(\log\;\Phi_{\text{MIP}}^{S}\right)$ rises in the task region compared with the pre-task region (Mann–Whitney $n_{1}=10314$, $n_{2}=32364$, moderate: $U=1.52\times 10^{8}$, $p<10^{-42}$, difficult: $U=1.52\times 10^{8}$, $p<10^{-39}$, easy: $U=1.55\times 10^{8}$, $p<10^{-29}$). We found significant differences in $z\left(\log\;\Phi_{\text{MIP}}^{S}\right)$ only between easy and moderate conditions (Kruskal-Wallis test: $\chi^{2}\left(2,N=32364\right)=12.2$, p=0.0022. Dunn’s multiple comparison test showed significant differences between easy and moderate condition, adjusted p=0.0015). However, we observed that the $z\left(\log\;\Phi_{\text{MIP}}^{S}\right)$ of the difficult condition showed significantly higher values than those of the moderate and easy conditions when τ was 1/500 s (see Figure S1).

Figure 2B shows the mean difference in the frequency rate of the MIP cut from the pre-task to the task. The MIP cut locations indicating significant changes are emphasized with asterisks. We found that the frequency rate of {EDA}-cut decreased and that of {ECG}-cut increased. This result indicates that the link between EEGs and EDA becomes stronger than that between EEGs and ECG. Interestingly, the frequency of the MIP cut location could not discriminate between the three different conditions {ECG}-cut in Figure 2B: one-way ANOVA, F(2,51)=0.142, p=0.868, $\eta^{2}=0.0024$; {EDA}-cut in Figure 2B: one-way ANOVA, F(2,51)=1.43, p=0.254, $\eta^{2}=0.028$). Unlike these indistinctive features of frequency rate, $z\left(\log\;\Phi_{\text{MIP}}^{S}\right)$ regarding {ECG} and {EDA} for the difficult condition showed significant differences compared with the other two conditions (Table 1).

**Table 1:** Average $z\left(\log\;\Phi_{\text{MI}P}^{S}\right)$, represented by $\left\langle z\left(\log\;\Phi_{\text{MI}P}^{S}\right)\right\rangle$, for each cut and its statistical result

MIP cut location	Kruskal-Wallis test	$\left\langle z\left(\log\;\Phi_{\text{MIP}}^{S}\right)\right\rangle$	Dunn’s multiple comparison test
ECG	$\chi^{2}\left(2,N=60884\right)=7.047$, p=0.03	Moderate: $7.43\times 10^{-2}$	M/E: p=0.734
		Easy: $7.02\times 10^{-2}$	M/D: p=0.265
		Difficult: $9.70\times 10^{-2}$	E/D: p=0.026
EDA	$\chi^{2}\left(2,N=24948\right)=14.95$, $p=10^{-4}$	Moderate: $1.98\times 10^{-1}$	M/E: p=0.843
		Easy: $2.13\times 10^{-1}$	M/D: $p<10^{-4}$
		Difficult: $2.65\times 10^{-1}$	E/D: p=0.009

### Main complex of the stress response

Next, we investigated the main complex of the entire system. The main complex comprises the main complexes (i.e., a subset of the entire set) and their $\Phi_{\text{MIP}}^{S}$. This section focuses on two kinds of $\Phi_{\text{MIP}}^{S}:\sum_{T\in C}z\left(\log\;\Phi_{\text{MIP}}^{T}\right)$ (i.e., the sum of $\Phi_{\text{MIP}}^{S}$ for all main complexes, a set S∈C. In short, it is represented by $\sum z\left(\log\;\Phi_{\text{MIP}}\right)$) and $\max_{T\in C}\;\left\{z\left(\log\;\Phi_{\text{MIP}}^{T}\right)\right\}$ (i.e., the maximum $z\left(\log\;\Phi_{\text{MIP}}^{T}\right)$ in a set C. Thus, we have $\max\left\{z\left(\log\;\Phi_{\text{MIP}}\right)\right\}$). Note that the system generally contains more than two main complexes.

**Figure 3:**
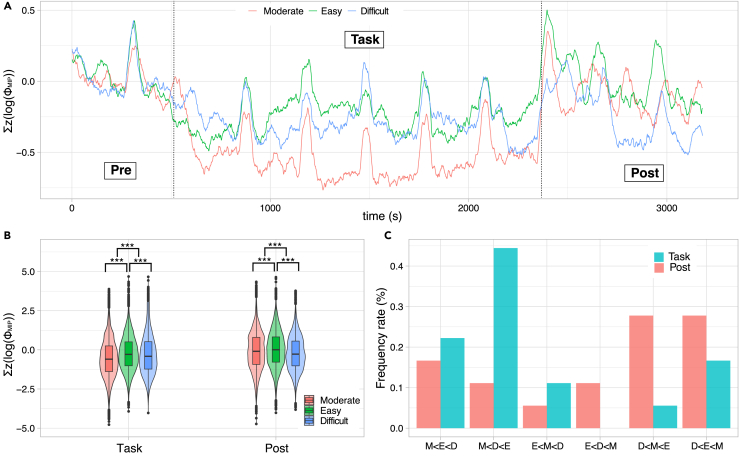
$\sum z\left(\log\;\Phi_{\text{MIP}}\right)$ for the three conditions (A) The time series of $\sum z\left(\log\;\Phi_{\text{MIP}}\right)$ for the three conditions applied is a moving average with 50 s. After the pre-task state (around 580 s), $\sum z\left(\log\;\Phi_{\text{MIP}}\right)$ drops its values for all conditions. (B) The $\sum z\left(\log\;\Phi_{\text{MIP}}\right)$ strength in the task and post-task phases. We confirmed the statistical difference for all pairs for Dunn’s multiple comparison test (Kruskal-Wallis test: $\chi^{2}\left(2,N=32364\right)=14.95$, $p=10^{-40}$). Box-and-whisker plots represent the median of the data (central thick line), the first and third quartiles (box), and 1.5× the interquartile range of the median (whisker). The shaded areas show violin plots of the data. (C) The frequency distribution for the mean $\sum z\left(\log\;\Phi_{\text{MIP}}\right)$ rank patterns for 18 individuals (see Figure S3 for each time series). We applied the Monte Carlo method to estimate the statistical differences under the uniform distribution assumptions (generate $10^{7}$ samples). The task condition is only significant (Task: $\chi_{Task}^{2}=13.3$, p=0.021; Post: $\chi_{Post}^{2}=4.67$, p=0.53). The disturbances (i.e., quasi-peaks) were observed every 500 s, resulting from the experimental protocol of saliva collection (note that hormonal data was not the target of this study).

Figure 3A shows the time series of $\sum z\left(\log\;\Phi_{\text{MIP}}\right)$ (see Figure S2 for the time series of $\max\left\{z\left(\log\;\Phi_{\text{MIP}}\right)\right\}$). $\sum z\left(\log\;\Phi_{\text{MIP}}\right)$ is the lowest for the moderate condition (Figure 3B). $\sum z\left(\log\;\Phi_{\text{MIP}}\right)$ of the easy condition is the highest. It is worth noting that the $\sum z\left(\log\;\Phi_{\text{MIP}}\right)$ in the moderate condition drops during the task phase. From an IIT perspective, this result is fascinating. The degree of integrity was lowest when the participant actively coped with stress. Furthermore, $\sum z\left(\log\;\Phi_{\text{MIP}}\right)$ for the difficult condition (considered the most stressed condition) was not the highest.

We examined the rank $\sum z\left(\log\;\Phi_{\text{MIP}}\right)$ during the task among the three conditions at each time step. The most frequent ranking was the easy, difficult, and moderate conditions in descending order (i.e., E > D > M in Figure 3C). The second-most frequent ranking was the difficult, easy, and moderate conditions in descending order (i.e., D > E > M in Figure 3C). Although individual differences were observed (Figure S3), the $\sum z\left(\log\;\Phi_{\text{MIP}}\right)$ in the moderate condition was consistently lower than those of the other two conditions (Monte Carlo method, p=0.021). Differences in the ranking may reflect how individuals cope with stress under various conditions, and these differences will be discussed in detail in the following section.

**Figure 4:**
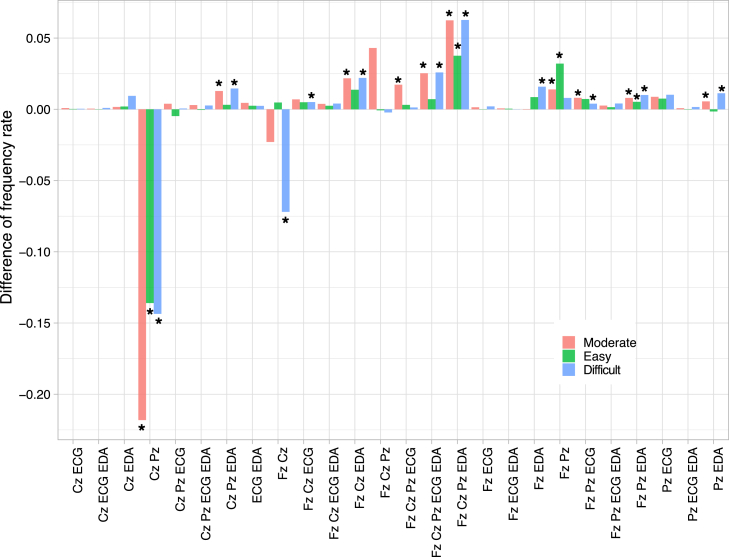
The mean frequency difference of the main complex between the pre-task and the task Freq(M|Task)−Freq(M|PreTask). The asterisk represents the statistically significant frequency compared with 0 (results from *t*-test if normative assumption is satisfied, otherwise, Mann–Whitney U test: p<0.05). Note that the asterisks are excluded when Freq(M|Task)−Freq(M|PreTask)<0.0005.

Figure 4 shows the frequency rate of main complexes with the highest value (i.e., $M={\mathrm{argmax}}_{T\in C}\;\left\{z\left(\log\;\Phi_{\text{MIP}}^{T}\right)\right\}$) in the task with respect to the pre-task. The number of {Cz, Pz} complexes significantly decreased under all conditions. In contrast, the number of complexes, including {EDA}, significantly increased. Interestingly, almost all the main complexes did not include the ECG. This observation highlights an increase of the {ECG}-cut, as depicted in Figure 2B. In terms of differences among conditions, the main complex comprising all components {Fz, Cz, Pz, ECG, EDA} shows a significant increase under moderate and difficult conditions but not in easy conditions. This differentiation may reflect the loss of agency in the easy condition. Specifically, in the easy condition, the main complex excludes EEG datasets, in contrast to the other conditions. This result suggests a dissociation of sympathetic nervous activity, estimated from EDA, as a part of the SAM-axis responses from the central brain information processing mechanisms.^45^^,^^46^ Conversely, in difficult and moderate conditions, the main complexes incorporate the SAM-axis responses more integrally. This observation matches some previous studies on the relationship between the sense of agency and SAM-axis response.^46^^,^^47^ Moreover, Table 2 shows significant differences among conditions in $\max\left\{\Phi_{\text{MIP}}^{M}\right\}$ of main complexes {Cz, Pz} and {Fz, Cz, Pz, EDA} of which frequency rates decreased and increased in all conditions, respectively (Figure 4).

**Table 2:** Averaged $\max\left\{z\left(\log\;\Phi_{\text{MI}P}^{M}\right)\right\}$

Maximum main complex	Kruskal-Wallis test	$\left\langle \max\left\{z\left(\log\;\Phi_{\text{MIP}}^{M}\right)\right\}\right\rangle$	Dunn’s multiple comparison test
Cz, Pz	$\chi^{2}\left(2,N=16023\right)=947$, $p=10^{-206}$	Moderate: $-4.46\times 10^{-1}$	M/E: $p<10^{-10}$
		Easy: $4.14\times 10^{-2}$	M/D: $p<10^{-10}$
		Difficult: $5.52\times 10^{-1}$	E/D: $p<10^{-10}$
Fz, Cz, Pz, EDA	$\chi^{2}\left(2,N=11137\right)=290.2$, $p=10^{-64}$	Moderate: $-9.82\times 10^{-1}$	M/E: $p<10^{-10}$
		Easy: $-6.15\times 10^{-1}$	M/D: $p<10^{-10}$
		Difficult: $-7.17\times 10^{-1}$	E/D: $p<10^{-4}$

We only listed the maximum main complex that showed significant differences for all conditions in Figure 4. See Table S1 for other cases of data duration.

### Individual differences in stress from the Integrated Information Theory perspective

We addressed individual differences in stress coping with each condition from the IIT perspective. Although IIT is likely to discriminate conditions, we also admit that the information structure of IIT (Figure 3C) varies among individuals. This difference in structure would be preferable when analyzing stress. It is well known that coping with stress differs among individuals because of genetic/epigenetic causes (e.g., hormone, neuroendocrine, and autonomic nervous system responses^11^). Stress coping also affects blood pressure and heart rate. These changes in the system are expected to affect subjective psychological states.^48^^,^^49^^,^^50^^,^^51^

If $\sum z\left(\log\;\Phi_{\text{MIP}}\right)$ reflects a subjective feeling, the subjective report would be associated with the given $\sum z\left(\log\;\Phi_{\text{MIP}}\right)$. We utilize $\sum z\left(\log\;\Phi_{\text{MIP}}\right)$ to evaluate the subjective report from IIT. As discussed in our previous study, $\sum z\left(\log\;\Phi_{\text{MIP}}\right)$ does not correlate with the subjective report itself.^41^ As the subjective report contains some biased issues, the difference from the baseline seems suitable for analysis.

In this section, we set the baseline of $\sum z\left(\log\;\Phi_{\text{MIP}}\right)$ as a moderate condition because we observed that $\sum z\left(\log\;\Phi_{\text{MIP}}\right)$ of the moderate condition was the lowest in the task (Figures 3A and 3B). We considered that the information structure of the moderate condition, related to active coping, was more stable than that of the other two conditions, related to passive coping. Therefore, the degree of subjective feeling is defined as follows:

$$s_{i}^{X}\left(\Phi_{\text{MIP}}\right)=\sum_{t=t_{1}}^{t_{2}}\left[\sum_{M\in M_{t}}\left\{z\left(\log\;\Phi_{\text{MIP},i}^{M,X}\right)-z\left(\log\;\Phi_{\text{MIP},i}^{M,\text{Moderate}}\right)\right\}\right]$$

where X is the easy or difficult condition and i is the index of the subject (we omit one individual sample because it contains many singular values of the pre-task phase for the IIT computation).

Following this definition, the subjective report was also adjusted in the easy/difficult condition with respect to the moderate condition. We denote this subjective rating of the subject i for each item I as $r_{i}^{I}=r_{\text{Task},i}^{I}-r_{\text{Pre},i}^{I}$. The *I* contains “exciting,” “effort,” “concentration,” “tiredness,” “boring,” “irritation,” and “annoying.” Therefore, the subjective rating minus the baseline value was $dr_{i}^{I,X}=r_{i}^{I,X}-r_{i}^{I,\text{Moderate}}$.

**Figure 5:**
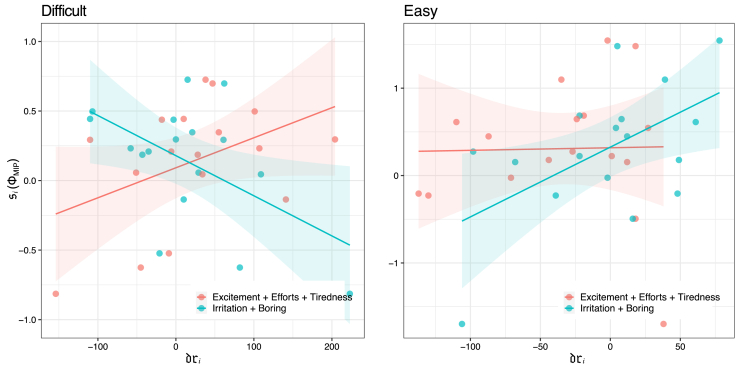
The correlation between $s_{i}^{X}\left(\Phi_{\text{MIP}}\right)$ and $dr_{i}$ for the difficult and easy condition $dr_{i}$ represents $dr_{i}^{\text{Excitement}}+dr_{i}^{\text{Effort}}+dr_{i}^{\text{Tiredness}}$ (i.e., positive valence) or $dr_{i}^{\text{Irritation}}+dr_{i}^{\text{Boring}}$ (i.e., negative valence). LEFT: The difficult condition for positive valence (red: Pearson’s correlation test: r=0.43,n=18,p=0.083) and negative valence (blue: Pearson’s correlation test: r=−0.53,n=18,p=0.029). RIGHT: The easy condition for positive valence (red: Pearson’s correlation test: r=0.15,n=18,p=0.63) and negative valence (blue: Pearson’s correlation test: r=0.63,n=18,p=0.008). The solid lines represent the linear fitting, and the shaded areas represent the 95% confidence intervals.

Figure 5 (left) shows the relationship between $s_{i}^{\text{Difficult}}\left(\Phi_{\text{MIP}}\right)$ and the subjective rating $dr_{i}^{\text{Difficult}}$ for the difficult condition. Note that a few relevant ratings were added up. The combinations shown in the figure indicated either the highest or the lowest correlation coefficients with $s_{i}^{\text{Difficult}}\left(\Phi_{\text{MIP}}\right)$ in all combinations. $s_{i}^{\text{Difficult}}\left(\Phi_{\text{MIP}}\right)$ positively correlated with positive valence item (Excitement, Effort, and Tiredness) and negatively correlated with negative valence item (Irritation and Boring). Figure 5 RIGHT shows the results regarding the same combinations of ratings in the easy condition. $s_{i}^{\text{Easy}}\left(\Phi_{\text{MIP}}\right)$ positively correlated with negative valence item. These results suggest that $s_{i}\left(\Phi_{\text{MIP}}\right)$ represents emotional effects in different ways according to the levels of task difficulty.

Specifically, the results of the correlation test between each item and $s_{i}\left(\Phi_{\text{MIP}}\right)$ are listed in Table 3. Only the item “Boring” has a significant correlation in both conditions, showing a negative correlation in the difficult condition and a positive correlation in the Easy condition. This observation has significant implications, such that boredom is associated with the entangled fluctuations of the system’s dynamics rather than a single physiological response. This understanding indicates that feelings of boredom should be viewed as complex interactions within the physiological system rather than isolated events. Moreover, (almost) significant correlation with the item “Boring” was consistent over different data duration $T_{d}=1000$ and $T_{d}=3000$ (see Table S2). For other items, we found that multiple items showed (almost) significant correlation only in difficult condition in the case of $T_{d}=1000$. In the easy condition, $s_{i}\left(\Phi_{\text{MIP}}\right)$ did not correlate to items with positive valence (excitement, effort, and concentration) in any case of data duration.

**Table 3:** Results of correlation test between $dr_{i}$ and $s_{i}\left(\Phi_{\text{MIP}}\right)$ for each item

Item	t	r	p
**Difficult**			

Excitement	1.91	0.44	0.08
Effort	0.84	0.21	0.41
Concentration	0.68	0.44	0.51
Tiredness	1.50	0.36	0.15
Irritation	−1.71	−0.40	0.11
Boring	−2.34	−0.52	**0.03**
Annoying	−1.64	−0.39	0.12

**Easy**			

Excitement	−0.16	−0.04	0.87
Effort	0.22	0.06	0.83
Concentration	−1.00	−0.25	0.33
Tiredness	0.77	0.20	0.45
Irritation	1.42	0.35	0.17
Boring	2.65	0.57	**0.02**
Annoying	1.17	0.29	0.26

Bold values show p<0.05.

### Conventional analyses of stress responses

Our experimental conditions had different difficulty levels of calculation tasks, i.e., easy < moderate < difficult. We investigated stress responses by means of conventional measures to verify if the calculation task functioned as a stressor. Physiological responses were estimated by heart rate (HR), skin temperature, and electrodermal activity (EDA). In addition, psychological responses were evaluated by ratings on visual analog scales with seven items.

**Figure 6:**
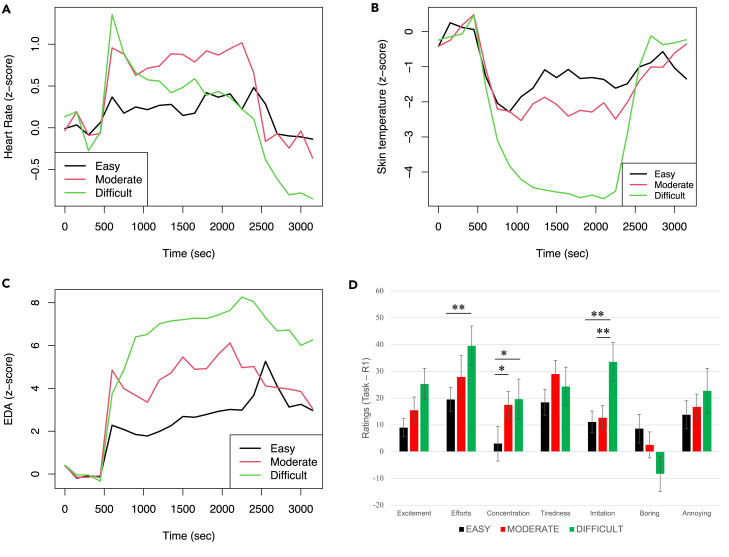
Results of conventional analyses to show psychophysiological stress responses (A–C) Heart rate, skin temperature, and electrodermal activity during experiment (mean per 150 s). 0-600s: pre-task rest phase; 600-2400s: task phase; 2400-3300s: post-task rest phase. (D) Changes of subjective ratings before and after the calculation task. Error bars indicate the standard error of the means. Asterisks indicate significant differences with multiple comparisons (∗: p<.05; ∗∗: p<.01). See Figure S5 for correlation coefficients in all pair of items in each condition, and Table S3 for correlation coefficients among psychological measures and physiological measures.

Figure 6A shows the average HR every 150 s. Note that the values were standardized (*Z* score) using the average and standard deviation of the pre-task rest period. A paired *t*-test was performed to test whether the average HR in the task period changed in comparison with the first rest period. Consequently, there was a significant increase in the moderate condition (t(17)=3.80,p=0.001), while there were no significant changes in other conditions (easy: t(18)=2.03,p=0.06; difficult: t(18)=1.77,p=0.09). Increased HR represents a typical acute stress response induced by a calculation task.^42^^,^^43^^,^^52^ Interestingly, the HR in the difficult condition rapidly increases at the beginning of the task period (At the first 150 s in difficult condition: t(18)=3.35,p=0.004) and then gradually decreases until the end of the task. This unstable change implies that participants engaged in the task as hard as they could at first, but they made less and less effort because the time limit was too short to do calculations.

Figure 6B shows the average skin temperature every 150 s. The skin temperature was represented by the relative temperature at the tip of the nose to the forehead. The data of one participant in the easy condition and one participant in the moderate condition were lost because of technical issues. The values were standardized (*Z* score), and the *t*-test was performed as in the HR case. The skin temperature significantly decreased during the task period in all conditions (easy: t(18)=3.36,p=0.003; moderate: t(18)=2.92,p=0.009; difficult: t(19)=3.08,p=0.006). The temperature at the tip of the nose has been used as an objective measure of acute stress responses,^43^^,^^53^^,^^54^^,^^55^^,^^56^^,^^57^ suggesting that the temperature decrease represents stress increase.

Figure 6C shows the averaged EDA every 150 s. The values were standardized (*Z* score), and the *t*-test was performed as in the case of the variables mentioned above. The EDA significantly increased during the task period in all conditions (easy: t(18)=4.66,p=0.0002; moderate: t(17)=3.78,p=0.002; difficult: t(19)=2.37,p=0.03), which signifies typical acute stress responses.^52^^,^^58^^,^^59^

Figure 6D shows differences in subjective ratings before and after the task. Results of a one-way ANOVA demonstrate significant differences among conditions in items Efforts (F(2,38)=4.51,p=0.02), Concentration (F(2,38)=4.67,p=0.02), and Irritation (F(2,38)=10.50,p=0.0002). We performed post-hoc comparisons with *t*-test corrected using an extended Bonferroni procedure on these scores. There is a significant difference between easy and difficult on item Efforts (t(19)=3.68,p=0.005). There are significant differences between conditions on item Concentration (easy vs. moderate: t(19)=3.35,p=0.01; easy vs. difficult: t(19)=2.47,p=0.02). There are significant differences between conditions on item Irritation (easy vs. difficult: t(19)=3.61,p=0.006; moderate vs. difficult: t(19)=3.28,p=0.006).

## Discussion

In this study, we verified the applicability of the IIT to physiological stress responses. There are three main results. First, the location of the MIP cut in the entire system during the task was fixed at the ECG instead of EDA. As a result, ECG data tends to being excluded from main complexes. These results suggest that cardiac activity and peripheral activity work in different information processes under stress.^16^^,^^17^^,^^18^ Second, $\sum z\left(\log\;\Phi_{\text{MIP}}\right)$ showed significant differences between conditions. The lowest $\sum z\left(\log\;\Phi_{\text{MIP}}\right)$ was found in the moderate condition, while higher values were found in the easy and difficult conditions. In addition, the main complex frequencies in the easy condition did not radically change between the pre-task and the task. This tendency is not observed in moderate and difficult conditions. Although $\sum z\left(\log\;\Phi_{\text{MIP}}\right)$ shows high values for easy and difficult conditions, the underlying information dynamics differ. Thus, the IIT differentiates the three conditions clearly by $\sum z\left(\log\;\Phi_{\text{MIP}}\right)$ and the frequency of the main Complex. Third, we have observed the correlation between subjective ratings $dr_{i}$ and the $\sum z\left(\log\;\Phi_{\text{MIP}}\right)$. We used the moderate condition, in which the lowest $\sum z\left(\log\;\Phi_{\text{MIP}}\right)$ was found, as a baseline. $\sum z\left(\log\;\Phi_{\text{MIP}}\right)$ demonstrated a negative correlation with negative valence, especially boredom, under difficult conditions and a positive correlation with them under easy conditions. In the context described, the IIT describes a neutral subjective feeling before the feeling is evaluated. This consideration matches the original definition of IIT on consciousness, which states $\Phi_{\text{MIP}}$ is a degree of awareness without its content.^35^ These findings are worth noting. When the same analyses were applied to individual physiological series (heart rate, skin temperature, and EDA), significant correlations of heart rate and skin temperature with some $dr_{i}$ were found only in the difficult condition and that of skin conductance was found only in the easy condition (see Table S3). This result highlights one of the major problems in stress study when subjective data are analyzed with a single physiological series: conflicts arise among multiple indicators. We must integrate information as a whole system to have a comprehensive view.

According to studies of stress responses in terms of hemodynamics, the elevation of blood pressure is induced by different mechanisms depending on the type of stressor.^44^^,^^60^ Participants actively cope with stressful tasks requiring them to be challenging and/or competitive. Conversely, participants passively cope with stressful tasks, keeping them from escaping and only allowing them to tolerate them. Active coping can evoke the elevation of blood pressure via increased cardiac output, whereas passive coping can produce it via increased peripheral vascular resistance.^61^^,^^62^ In the present study, changes in the skin temperature and the EDA, which are associated with peripheral sympathetic nervous system activity, indicated stress responses in all conditions. However, changes in the HR, which is associated with cardiac sympathetic nervous system activity, indicated stress responses only in moderate conditions. These results suggest that participants adopted active coping in moderate conditions and passive coping in easy and difficult conditions. Moreover, the skin temperature and the EDA seem to have different degrees of physiological stress response among conditions, i.e., easy < moderate < difficult, although there are no significant differences. Therefore, passive coping in difficult conditions might be qualitatively different from passive coping in easy conditions. In fact, according to the psychological responses, participants felt differences in effort, concentration, and irritation between those conditions. Results of IIT in this study can provide greater insight into the qualitative difference between easy and difficult conditions.

Danckert and Eastwood’s discussion of boredom helps us understand the close relationship between subjective scores and non-valence ($\Phi_{\text{MIP}}$). They stated that boredom should satisfy two factors: disengagement and loss of agency.^25^^,^^63^ Based on their definition, we can classify the three tasks as follows: the moderate condition satisfies neither of the factors because the participant could engage in the task at their own pace; the difficult condition satisfies only the disengagement factor because the participant performed the task despite its passiveness; and the easy condition satisfies both the factors both because the participant could not focus on the task, and their attention inevitably diverged to the other unconstrained factors. This classification is also observed in our IIT analysis. The dissociation of the SAM-axis response for the easy condition indicates the loss of agency^45^^,^^46^^,^^47^ (Figure 4). Contrarily, $\Phi_{\text{MIP}}$ represents the participant’s disengagement from the task, as the highly entangled information processes hinder the participant’s concentration. The increased complexity in information processing may detract from the participant’s ability to focus effectively on the task at hand. Hence, Danckert and Eastwood’s classification can explain why the easy condition positively correlated with the boring score, while the difficult condition correlated negatively (Figure 5). The sense of agency in the task prevented subjective feelings of boredom.^63^^,^^64^ Instead, the participant felt the task was not boring and attempted to allocate different affections to their affective state reflectively.

Our stressor-free estimation method for stress sheds light on an aspect of the hidden structure of subjective feelings. In the free energy principle (FEP) framework, the changing free energy rate can explain the affection.^65^^,^^66^ This framework can construct a definite objective relation between subjective affection and the system process. In contrast, the subject allocates its subjective values concerning $\Phi_{\text{MIP}}$ (i.e., entangled system fluctuation) in the IIT framework. From this subject relation to the objective physiological state, we can expect some law-like relation in the subjective judgment through IIT. As we have observed, there is a non-trivial relationship between boredom and other subjective scores. The boredom feeling (i.e., the positive correlation with $s_{i}\left(\Phi_{\text{MIP}}\right)$) never coexists with the other positive valences (Figure 5 RIGHT), while the non-boredom feeling (i.e., the negative correlation with $s_{i}\left(\Phi_{\text{MIP}}\right)$) coexists the other positive valences (Figure 5 LEFT). Interestingly, this asymmetric relationship on valence allocations matches the classical observation on boredom, which states that a highly bored person is not good at labeling their affective state.^67^^,^^68^^,^^69^ By contrast, the boredom score $dr_{i}^{\text{Boring}}$ itself never shows the statistical difference (easy: 4.35 ± 29.5. difficult: −10.2 ± 35.9. Paired *t*-test: t(16)=1.60, p=0.12). If we only consider the boredom score as a subjective rating, there is no difference between the two conditions. Therefore, our method discriminates the differences between the two conditions.

However, defining $\Phi_{\text{MIP}}$ as disengagement contextualizes its meaning. This contextuality is not unique to $\Phi_{\text{MIP}}$, determining what subjective meanings $\Phi_{\text{MIP}}$ might have remained problematic. Our method does not argue that $\Phi_{\text{MIP}}$ itself is a measure of subjectivity; rather, $\Phi_{\text{MIP}}$ serves as a measure of the entangled system fluctuation, and subjective evaluation is a reflective judgment of this value. For example, considering the correlation between $\Phi_{\text{MIP}}$ and the rubber hand illusion,^41^ complex entangled physiological information processes can distort a natural action-perception structure.^70^^,^^71^ We, therefore, propose that $\Phi_{\text{MIP}}$ (i.e., the entangled system fluctuation) reflects subjective feelings that cannot be directly linked to specific actions. In other words, the entangled system fluctuation hinders the subject’s engagement, weakening the link between behavior and primary subjective feeling,^72^ leading to higher-order emotions such as boredom. We claim that $\Phi_{\text{MIP}}$ are subjective feelings that cannot be reduced to a specific action rather than stress itself. Note, however, that this disengagement state is uncontrollable.^9^ We still leave room for $\Phi_{\text{MIP}}$ to correlate with subjective stress. It should be emphasized that our interpretations remain speculative. Applying IIT to physiological data has the advantage of avoiding latent variable problems, although challenges to its interpretations remain.

In summary, $\sum z\left(\log\;\Phi_{\text{MIP}}\right)$ represents the feeling of being subjected to the entangled system fluctuation. $\sum z\left(\log\;\Phi_{\text{MIP}}\right)$ bridges the subjective and objective states. Furthermore, $\sum z\left(\log\;\Phi_{\text{MIP}}\right)$ represents the non-valence feeling. Participants labeled their feelings according to their degree of agency. Without this agency, the participants tended to evaluate the “bored” state. Our interpretation remains speculative because no evidence supports this affective allocation process. However, it seems true that $\sum z\left(\log\;\Phi_{\text{MIP}}\right)$ reflects some aspects of subjective feelings through the objective system process.

### Limitations of the study

Although we found that $\sum z\left(\log\;\Phi_{\text{MIP}}\right)$ can measure subjective stress, and whether this correspondence is valid remains uncertain. This correspondence assumes that the entangled system fluctuation generates subjective discomfort. Furthermore, our method needs a baseline $\sum z\left(\log\;\Phi_{\text{MIP}}\right)$ (moderate condition, in this study). In general, there is no guarantee that such a baseline will be obtained. Furthermore, in this study, disturbances in physiological data were made by the saliva collection. The disturbances overlapped with normal EEG and could hardly be removed by signal processing filters. However, saliva collection was performed at precisely the same time for all participants and conditions (within-subjects design), so statistical comparisons between conditions are possible. Therefore, we employed the raw EEG data, including the disturbances associated with the collection. Future studies should carefully avoid such disturbances to address this concern.

## STAR★Methods

### Key resources table

**Table:** 

REAGENT or RESOURCE	SOURCE	IDENTIFIER
**Deposited data**		

Data and statistical analyses	This paper	https://github.com/t-niizato/Stress-without-a-stressor-Integrated-information-theory-explains-the-information-dynamics-of-stress

**Software and algorithms**		

MATLAB (v. 2022b)	MathWorks	https://www.mathworks.com/
Practical PHI Toolbox	Oizumi et al.^34^	https://figshare.com/articles/code/phi_toolbox_zip/3203326
R	R Core Team	https://www.r-project.org

### Resource availability

#### Lead contact

Further information and requests for resources should be directed to and will be fulfilled by the Lead Contact, Takayuki Niizato (niizato@iit.tsukuba.ac.jp).

#### Materials availability

This study did not generate new unique reagents.

#### Data and code availability

• **Data:** All data has been deposited at Github and is publicly available as of the date of publication at https://github.com/t-niizato/Stress-without-a-stressor-Integrated-information-theory-explains-the-information-dynamics-of-stress.
• **Code:** All original code has been deposited at Github and is publicly available as of the date of publication at https://github.com/t-niizato/Stress-without-a-stressor-Integrated-information-theory-explains-the-information-dynamics-of-stress.
• **Additional information:** Any additional information required to reanalyze the data reported in this paper is available from the lead contact upon request.

### Experimental model and study participant details

#### Ethics statement

Ethics Committee of Nagaoka University of Technology approved the study (Approval Number: H28-8). All participants provided written informed consent prior to the experiment after the procedures involved were explained.

#### Study participant details

Twenty volunteers (5 women and 15 men, 22–30 years old, Japanese) were recruited from among the students at Nagaoka University of Technology. The participants were medically healthy based on physical examination and had no history of taking drugs, endocrine medicine, chronic trauma, or other chronic diseases. Those who currently used medication for anxiety, chronic depression disorders, asthma, or minor illnesses were excluded from the experiments. The participants were also forbidden from drinking alcohol, smoking, or consuming sleeping pills on the day of the experiment.

### Method details

#### Procedure

The participants performed three calculation tasks on different days in random order, a within-subject design. Experiments were carried out in a quiet room with the temperature at 22°C. The subjects sat on a soft, comfortable sofa equipped with devices for physiological measurements. The experiment lasted 55 min: 10 min of pre-task rest, 30 min of calculation task, and 15 min of post-task rest. Physiological measurements, EEG, ECG, EDA, and also skin temperature measurements were performed throughout the experiment. Subjective reports using the visual analogue scale were obtained at the end of the pre-task rest, task, and post-task rest. We also collected saliva every 5 min, although the hormone test was not carried out because we focused on electrophysiological time-series data in this study.

#### Experimental conditions

This experiment investigated the acute stress response to three different difficulty levels in the calculation task. The participants performed a continuous arithmetic task on a computer screen using a keyboard. They were instructed to complete it as fast and precisely as possible, according to the following conditions.

##### Easy condition

The participants responded to single-digit addition problems by pressing numeric keys for 3 s per problem. The questions were automatically updated every 3 s. This time restriction was sufficient to input the answer. Through this design, we expected that the participants could perform the task with spare time and, therefore, trigger boredom. Thus, this condition can be regarded as a passive coping task, as the participants could not control the progress of the task.

##### Moderate condition

The participants answered single-digit addition problems by pressing numeric keys as quickly as possible. The questions were updated when the participants pressed the Enter key. We expected that the participants would be able to perform the task actively at their own pace without any time restrictions. Thus, this condition can be regarded as an active coping task as the participants could control the progress of the task.

##### Difficult condition

The participants answered double-digit addition/subtraction problems by pressing numeric keys for 3 s per problem. The questions were updated every 3 s. This time restriction was too short to input the answers correctly. Therefore, we expected the participants to find it difficult to answer. Thus, this condition can be regarded as a passive coping task because the participants could not control the progress of the task, as in the Easy condition.

#### Physiological measurement

Physiological measurements for EEG, ECG, and EDA were performed using a bio-signal amplifier system (Polymate AP216, Miyuki Giken Co. Ltd., Japan) with 16-bit resolution and a 500 Hz sampling rate. Electrodes for EEG were placed at Fz, Cz, and Pz with references (A1 and A2) as per the standardised 10–20 international system. We intended to explore a wide range of brain activity with a small number of electrodes to reduce participants’ burden. The EEG signals were filtered with a high (30 Hz) and low (1 Hz) cut filter and a 50 Hz notch filter. Electrodes for ECG were placed under the right clavicle and on the lower left abdomen (the so-called “Lead II” induction). Electrodes for EDA were placed on the palmar side of the middle phalanges of the second and fourth fingers of the participant’s non-dominant hand. The ECG and EDA signals were filtered using a high (30 Hz) cut filter and a 50 Hz notch filter.

The skin temperature at the tip of the nose, with a reference to the forehead, was recorded using a thermistor probe (ITP082-25, NIKKISO-THERM Co., Ltd., Japan) and a data logger (NT Logger N543, NIKKISO-THERM Co., Ltd., Japan) at a sampling rate of 1.0 Hz.

#### Psychological measurement

A questionnaire consisting of seven items, “exciting,” “effort,” “concentration,” “tiredness,” “boring,” “irritation,” and “annoying,” was administered. We assumed that these items independently represented participants’ emotional affects (see Figure S5 for correlation matrices among items). The participants indicated their responses on a visual analogue scale (VAS) ranging from 0 (do not feel so at all) to 100 (very strong feeling). The standardised questionnaire, such as Profile of Mood States (POMS),^73^ takes much time to respond to all given items; occasionally, it turns into a methodological limitation. Comparatively, the VAS, which we used in this study, is simple and easy to respond to, e.g., it took less than 10 s, and it should be useful as far as measuring “relative” values in cases of measuring multiple times in a within-subject study.

### Quantification and statistical analysis

#### Basic concept of IIT

Although many concepts exist in IIT, we focus on three aspects: the minimum information partition (MIP), main complexes, and integrated information Φ. IIT deals with intrinsic rather than extrinsic information; it depends only on inner variables. Typical biological information theories focus on the relationship between external inputs and their results. Under this setting, the system becomes a black box. IIT aims to consider this black box in terms of “what the system is” rather than “what the system does”.^37^ The basic concept of IIT expresses the mutual information between the past and current system states, which is mathematically expressed as I(X(t−τ);X(t)). However, this simple mutual information contains redundant information on the system’s integrity, which should be measured as the irreducibility of the system into its parts (subsystems). The information obtained from the subsystems must be subtracted from all the information I(X(t−τ);X(t)). The remaining information represents the system’s integrity; therefore, the problem of IIT lies in determining system partitions.

This study applies IIT as the mismatched decoding method proposed by Oizumi et al.^34^ because $\Phi^{*}$ satisfies the following properties: (i) non-negativity: the lower bound of $\Phi^{*}$ is 0, (ii) sensitivity to noise correlation: $\Phi^{*}$ deals with external, correlated noise,^36^ and (iii) applicability to continuous variables. Note that $\Phi^{*}$ is a natural extension of the discrete Φ. The IIT as the mismatched decoding is sometimes classified as “IIT 2.0” to distinguish it from IIT 3.0.^34^^,^^35^^,^^37^ In this study, we did not apply IIT 3.0 to the physiological data because IIT 3.0 deals only with discrete values.

#### Integrated information ($\Phi^{*}$) and MIP

As mentioned in the previous section, the partitioning mode is essential for determining the system’s integrity. MIP is a system partition in which the system’s integrity is minimal. The integrity of the system in $\Phi^{*}$ is given by

$$\Phi^{*}=I\left(X\left(t-\tau \right);X\left(t\right)\right)-I^{*}\left[X;\tau ,\pi \in P_{S}\right]$$

where I(X(t−τ);X(t)) is the mutual information between the current X(t) and past states X(t−τ); S is the set of all nodes of a given system; $P_{S}$ is the set of all bi-partitions (total $2^{\left|S\right|-1}$ partitions); $I^{*}\left[X;\tau ,P_{S}\right]$ is a “hypothetical” mutual information, indicating the mismatched decoding in the partitioned probability distribution. More precisely, $I^{*}\left[X;\tau ,\pi \in P_{S}\right]$ is given as the partition $\max_{\beta }\tilde{I}\left(\beta ;X,\tau ,\pi \in P_{S}\right)$ that minimises $\Phi^{*}$ (see listed studies^34^^,^^36^ for further details about this expression).

As $\Phi^{*}$ depends on the partition $\pi \left(\in P_{S}\right)$, MIP is the partition that minimises the integrated information.

$$\pi_{\text{MIP}}≔\arg\min_{\pi \in P_{S}}\Phi^{*}\left(\pi \right)$$

The integrated information for $\pi_{\text{MIP}}$ is expressed as $\Phi^{*}\left(\pi_{\text{MIP}}\right)$. We simply denote $\Phi^{*}\left(\pi_{\text{MIP}}\right)$ as $\Phi_{\text{MIP}}$. Notably, if $\Phi_{\text{MIP}}$ equals 0, the parts of the system are mutually independent; that is, there is no interaction between the parts. In this sense, $\Phi_{\text{MIP}}$ characterises the irreducibility of a system into its parts.

#### Main complex

In general, we can compute $\Phi_{\text{MIP}}$ for any subsystem in the system, not only for the set S. We denote each $\Phi_{\text{MIP}}$ for a subsystem T as $\Phi_{\text{MIP}}^{T}$, where T⊂S. A “complex” is a subsystem C(⊂S), where $\Phi_{\text{MIP}}^{C}>\Phi_{\text{MIP}}^{T}$ for all supersets of S. Based on this definition, we define the main complexes as those with a local maximum $\Phi_{\text{MIP}}^{M}$. Therefore, a main complex is a complex M satisfying $\Phi_{\text{MIP}}^{M}>\Phi_{\text{MIP}}^{R}$ for the subsystem R⊂M. This definition states that they are exclusive if two main complexes exist (say, A and B). IIT researchers consider these main complexes to be the core of the system’s information, which may be related to our conscious experience.

#### Data setting for IIT 2.0 application

Oizumi et al. have proposed approximation methods for the computational problem of IIT.^34^ This study applied their “Practical Φ Toolbox for MATLAB” to the physiological data; we considered only five types of physiological data for the eighteen participants. Note that two of the twenty participants were excluded from this analysis as at least one of their physiological data was missing for technical reasons during trials. The exhaustive method (computing all possible minimum information partitions [MIPs]) can be applied within a realistic computation time for such a small system (Figure 1A).

Another parameter is the time delay τ (IIT has only two constraints: partition π and time delay τ). We computed $\Phi_{\text{MIP}}$ for τ from 0 to 500 frames to choose the suitable parameter. The time delay τ is the point at which the mean is the maximum $\Phi_{\text{MIP}}$ for all the subjects. This τ was approximately 50 frames (i.e., 0.1 s, see Figure S4). This value seems suitable because it is the same as the human response time.

The time series of each set of raw data was from frames A to B (500 fps), and as we applied a 50-frame delay (0.1 s) for the computation of IIT 2.0, a sufficient data duration $T_{d}$ was required for IIT 2.0. We attempted $T_{d}$ = 1000 frames (2 s), 2000 frames (4 s), and 3000 frames (6 s) for the IIT 2.0 computations---the time window shifted by 500 frames each. The first set of blank data was set to zero (only the first six steps, at most, for 3000 frames), which sum the A to B datasets for each participant.
