# Supplementary material for: Toward stressor-free stress estimation: The integrated information theory explains the information dynamics of stress
Source: iScience. 2024 Jul 26;27(8):110583. doi: 10.1016/j.isci.2024.110583 (PMC11357877; doi:10.1016/j.isci.2024.110583)
Supplement: Document S1. Figures S1–S5 and Tables S1–S3 [file mmc1.pdf]

iScience, Volume *27*

Supplemental information

Toward stressor-free stress estimation: The integrated information theory explains the information dynamics of stress

Takayuki Niizato, Yuta Nishiyama, Yuta Oka, Poe Thinzar Aung, and Shusaku Nomura

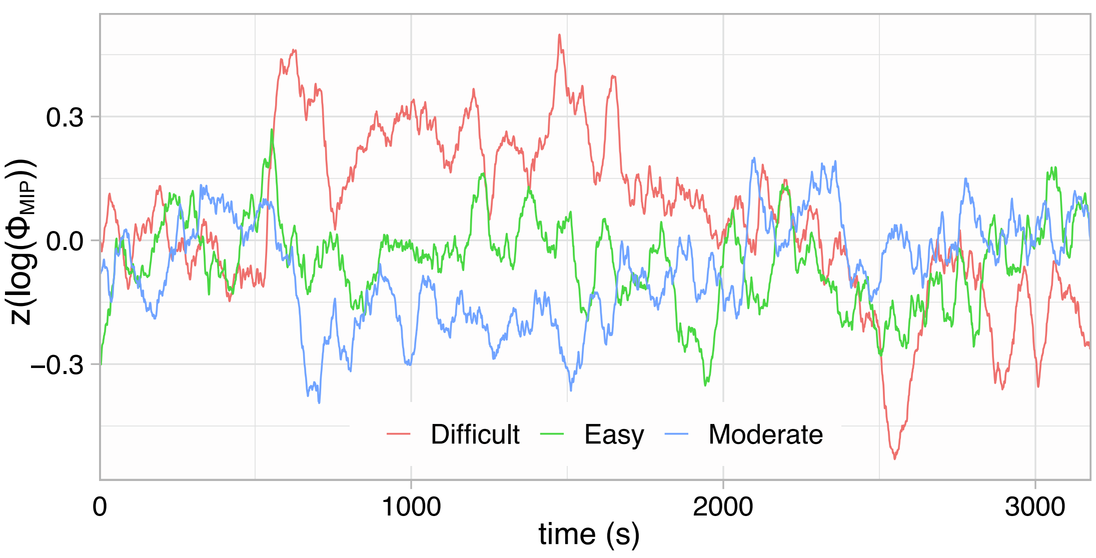

Figure S1: $z\left(\log \Phi_{\mathrm{MIP}}^{S}\right)$ **with time delay** $\tau = 1/500$ **for the entire system** S **for each condition, related to Figure 2A.**

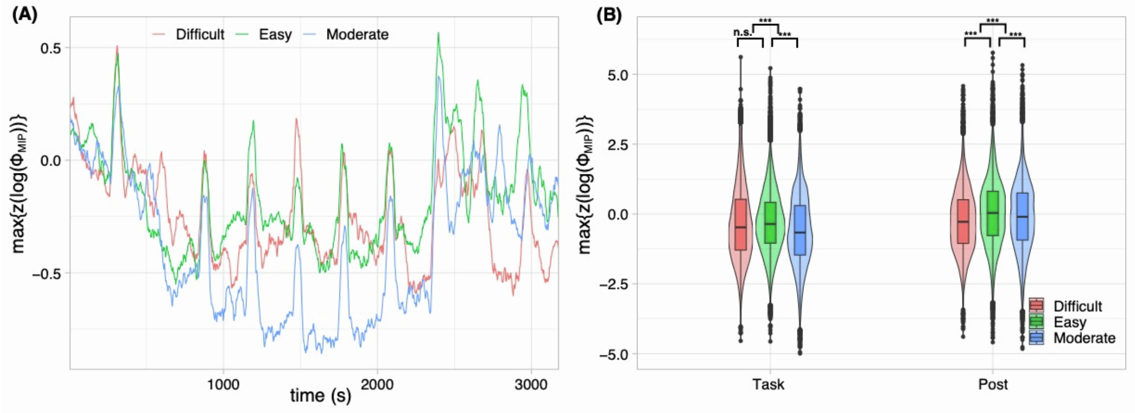

Figure S2: $\max\{z(\log \Phi_{\mathrm{MIP}})\}$ **for the three conditions, related to Figure 3.** (A) Time series of $\max\{z(\log \Phi_{\mathrm{MIP}})\}$ with time delay $\tau = 0.1$ (s) (50-s moving average). (B) The $\max\{z(\log \Phi_{\mathrm{MIP}})\}$ strength in the task phase and the post-task phase.

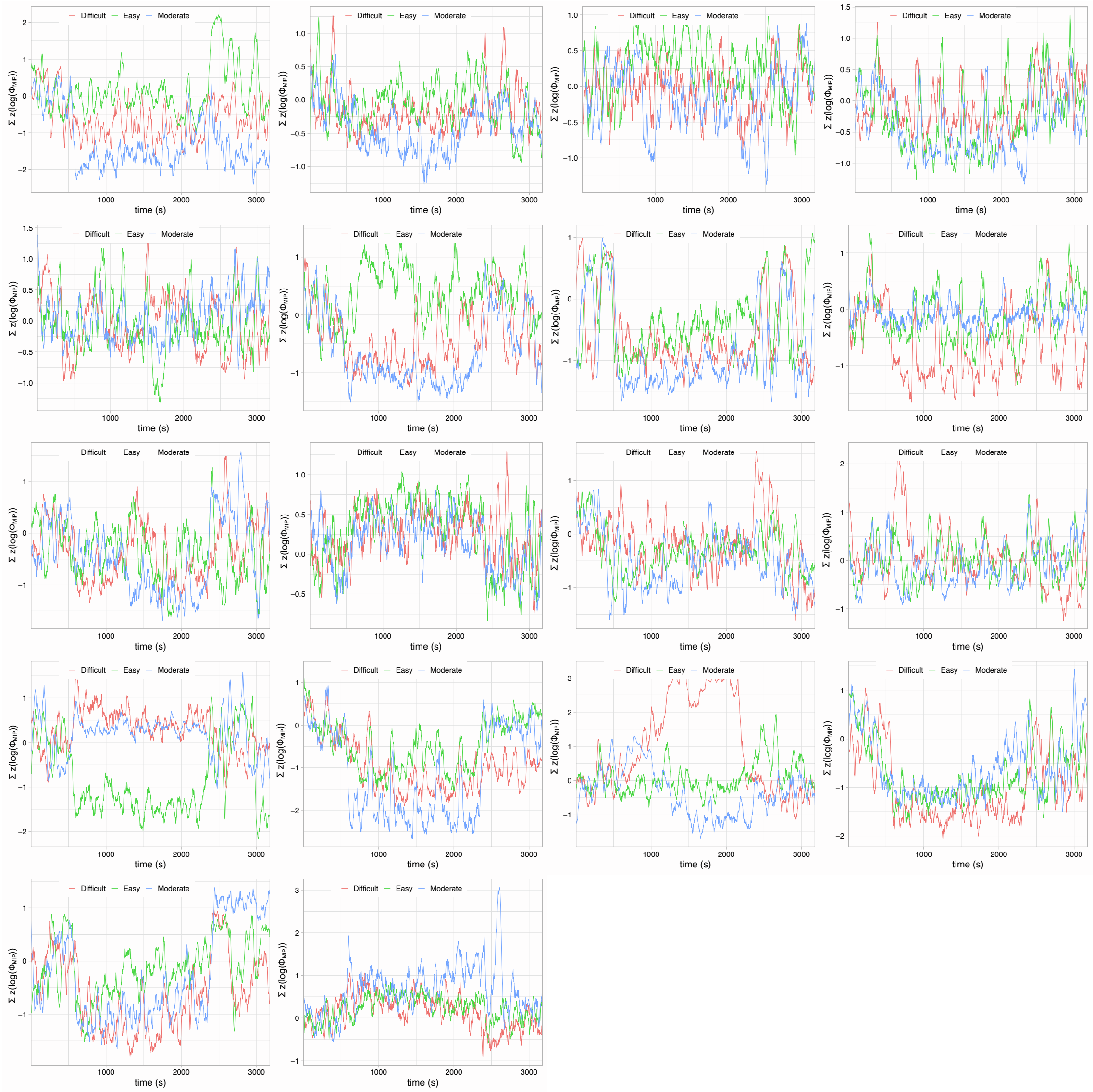

Figure S3: $\sum z(\log \Phi_{\mathrm{MIP}})$ **with time delay** $\tau = 0.1$ **(s) for each participant, related to Figure 3A.**

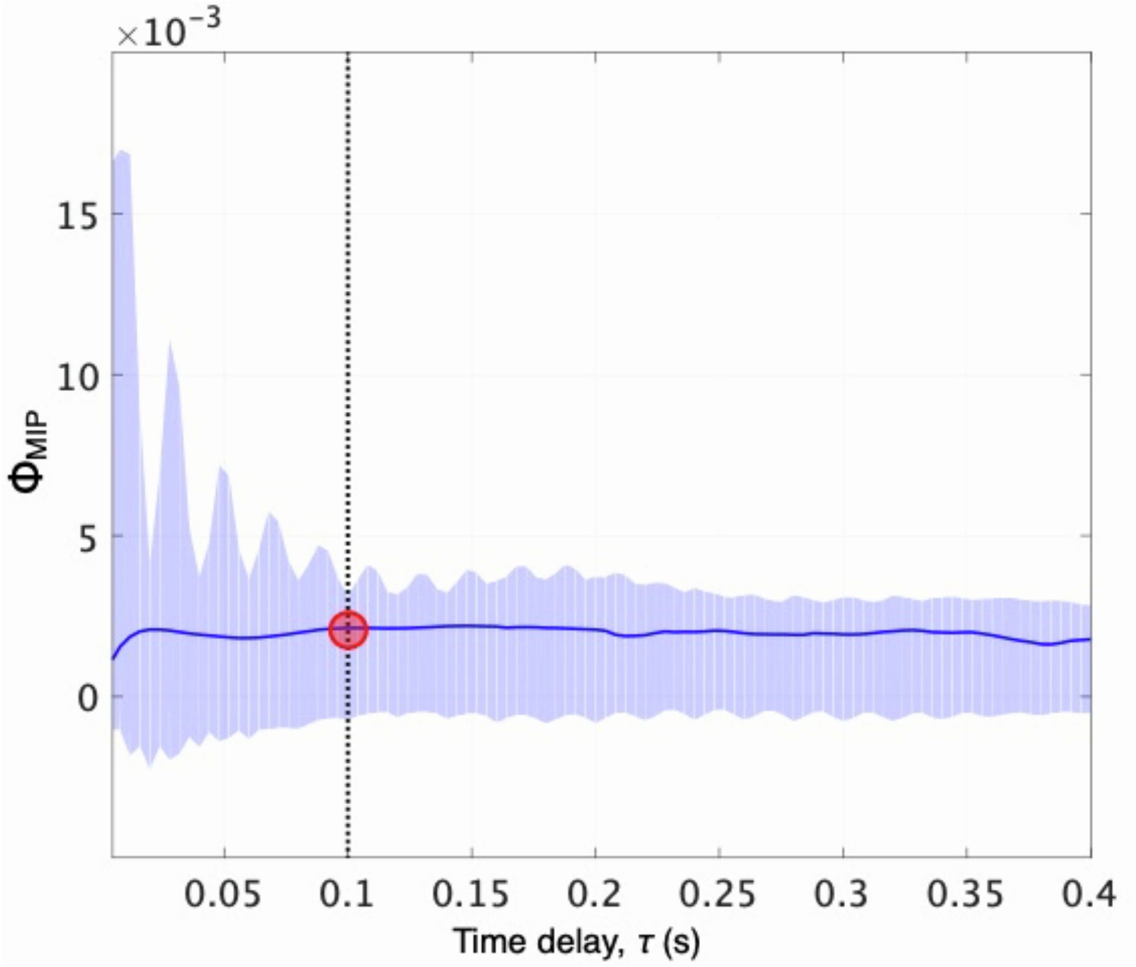

Figure S4: Φ_{MIP} along time delay τ for the entire system S.The Φ_{MIP} peak is located at 0.1 s, related to STAR Methods.

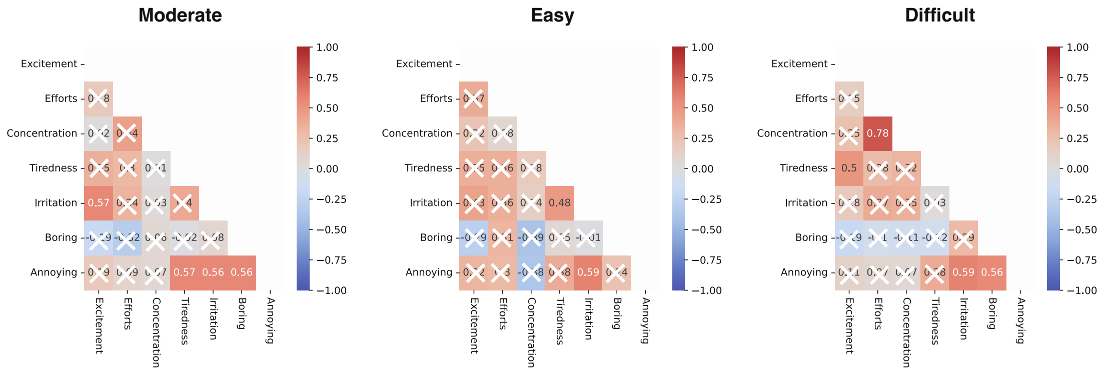

Figure S5: Correlation matrix for each condition, with the colour bar indicating the correlation coefficient values, related to Figure6. Crosses denote non-significant results from Pearson's correlation test.

Table S1: Averaged $\max\{z(\log \Phi_{\mathrm{MIP}}^{M})\}$ when the data duration $T_d = 1000, 3000$, related to Table 2.

$T_d = 1000$

Maximum main complex	Kruskal-Wallis test	$\langle \max\{z(\log \Phi_{\mathrm{MIP}}^{M})\} \rangle$	Dunn's multiple comparison test
Cz, Pz	$\chi^2(2, N = 10080) = 947$, $p = 10^{-143}$	Moderate: -1.28×10^{-1}<br>Easy: 2.43×10^{-1}<br>Difficult: 8.86×10^{-1}	M/E: $p < 10^{-10}$<br>M/D: $p < 10^{-10}$<br>E/D: $p < 10^{-10}$
Fz, Cz, Pz, EDA	$\chi^2(2, N = 12486) = 656$, $p = 10^{-6}$	Moderate: -3.90×10^{-1}<br>Easy: -2.98×10^{-1}<br>Difficult: -3.12×10^{-1}	M/E: $p < 10^{-6}$<br>M/D: $p = 0.057$<br>E/D: $p = 0.039$

$T_d = 3000$

Maximum main complex	Kruskal-Wallis test	$\langle \max\{z(\log \Phi_{\mathrm{MIP}}^{M})\} \rangle$	Dunn's multiple comparison test
Cz, Pz	$\chi^2(2, N = 19867) = 1022$, $p = 10^{-222}$	Moderate: -6.50×10^{-1}<br>Easy: 3.46×10^{-1}<br>Difficult: 6.65×10^{-2}	M/E: $p < 10^{-10}$<br>M/D: $p < 10^{-10}$<br>E/D: $p < 10^{-10}$
Fz, Cz, Pz, EDA	$\chi^2(2, N = 9593) = 529$, $p = 10^{-115}$	Moderate: -1.35×10^{-1}<br>Easy: -7.55×10^{-1}<br>Difficult: -9.89×10^{-1}	M/E: $p < 10^{-10}$<br>M/D: $p < 10^{-10}$<br>E/D: $p < 10^{-4}$

Table S2: Results of correlation test between $\mathfrak{dr}_i$ and $\mathfrak{s}_i(\Phi_{\mathrm{MIP}})$ for each item in cases of data duration $T_d = 1000, 3000$, related to Table 3. Bold values show $p < 0.1$.

$T_d = 1000$		
**Item**	r	p
Difficult		
**Excitement**	**0.60**	**0.012**
**Effort**	**0.42**	**0.092**
**Concentration**	**0.43**	**0.085**
Tiredness	0.35	0.16
Irritation	–0.41	0.99
**Boring**	**–0.78**	**0.00**
**Annoying**	**–0.64**	**0.005**
Easy		
Excitement	0.06	0.83
Effort	0.11	0.97
Concentration	–0.24	0.36
Tiredness	0.13	0.61
Irritation	0.33	0.20
**Boring**	**0.47**	**0.057**
Annoying	0.20	0.45

$T_d = 3000$		
**Item**	r	p
Difficult		
Excitement	0.60	0.22
Effort	0.42	0.55
Concentration	0.43	0.72
Tiredness	0.35	0.28
Irritation	–0.41	0.15
**Boring**	**–0.78**	**0.095**
Annoying	0.64	0.23
Easy		
Excitement	–0.08	0.78
Effort	0.07	0.79
Concentration	–0.24	0.35
Tiredness	0.20	0.44
Irritation	0.31	0.22
**Boring**	**0.58**	**0.014**
Annoying	0.30	0.24

Table S3: Correlation analysis between $\mathfrak{dr}_i$ and each physiological dataset, related to Figure 6. Bold values show $p < 0.05$.

	Condition	Questionnaire Item	df	*t*	*p*	*r*
Heart Rate	Easy	Excitement	16	1.2	0.25	0.29
		Efforts	16	0.69	0.5	0.17
		Concentration	16	0.49	0.63	-0.12
		Tiredness	16	0.01	0.99	0.002
		Irritation	16	0.42	0.68	0.1
		Boring	16	0.8	0.43	0.2
		Annoying	16	0.71	0.49	-0.17
		Excitement+Efforts+Tiredness	16	1.09	0.29	0.26
		Irritation+Boring	16	0.86	0.4	0.21
	Difficult	Excitement	16	1.66	0.12	0.38
		**Efforts**	**16**	**2.52**	**0.02**	**0.53**
		Concentration	16	0.66	0.52	0.16
		Tiredness	16	0.61	0.55	0.15
		Irritation	16	1.49	0.16	-0.35
		Boring	16	0.99	0.34	-0.24
		Annoying	16	1.74	0.1	-0.4
		**Excitement+Efforts+Tiredness**	**16**	**2.3**	**0.04**	**0.5**
		Irritation+Boring	16	1.53	0.15	-0.36
Skin Temperature	Easy	Excitement	16	1.51	0.15	-0.35
		Efforts	16	1.33	0.2	0.32
		Concentration	16	0.25	0.8	-0.06
		Tiredness	16	1.25	0.23	0.3
		Irritation	16	0.75	0.46	-0.18
		Boring	16	0.1	0.92	-0.03
		Annoying	16	0.33	0.75	0.08
		Excitement+Efforts+Tiredness	16	0.65	0.53	0.16
		Irritation+Boring	16	0.4	0.69	-0.1
	Difficult	**Excitement**	**17**	**2.68**	**0.02**	**-0.54**
		Efforts	17	1.15	0.27	-0.27
		Concentration	17	1.77	0.09	-0.39
		Tiredness	17	1.13	0.28	-0.26
		Irritation	17	0.32	0.75	-0.08
		Boring	17	2.06	0.06	0.45
		Annoying	17	1.76	0.1	0.39
		**Excitement+Efforts+Tiredness**	**17**	**2.3**	**0.03**	**-0.49**
		Irritation+Boring	17	1.13	0.27	0.26
EDA	Easy	Excitement	15	1.07	0.3	0.27
		Efforts	15	0.67	0.51	0.17
		Concentration	15	2.03	0.06	0.46
		**Tiredness**	**15**	**2.14**	**0.05**	**-0.48**
		Irritation	15	0.33	0.75	-0.08
		**Boring**	**15**	**4.11**	**0.0009**	**-0.73**
		Annoying	15	1.99	0.07	-0.46
		Excitement+Efforts+Tiredness	15	0.1	0.92	-0.03
		**Irritation+Boring**	**15**	**3.31**	**0.005**	**-0.65**
	Difficult	Excitement	16	1.06	0.31	0.26
		Efforts	16	1.05	0.31	0.25
		Concentration	16	0.93	0.37	0.23
		Tiredness	16	0.51	0.61	0.13
		Irritation	16	0.78	0.44	-0.19
		Boring	16	0.05	0.96	-0.01
		Annoying	16	0.13	0.89	-0.03
		Excitement+Efforts+Tiredness	16	1.27	0.22	0.3
		Irritation+Boring	16	0.42	0.68	-0.11
